# The Effects of Urinary Albumin and Hypertension on All-Cause and Cardiovascular Disease Mortality in Korea

**DOI:** 10.1093/ajh/hpx051

**Published:** 2017-05-02

**Authors:** Mi Hae Seo, Jong-Young Lee, Seungho Ryu, Yu Sam Won, Ki Chul Sung

**Affiliations:** 1 Department of Internal Medicine, Soonchunhyang University Gumi Hospital, Gumi, Korea;; 2 Division of Cardiology, Department of Medicine, Kangbuk Samsung Hospital, Sungkyunkwan University School of Medicine, Seoul, Korea;; 3 Department of Occupational and Environmental Medicine, Kangbuk Samsung Hospital, Sungkyunkwan University, School of Medicine, Seoul, Korea;; 4 Department of Neurosurgery, Kangbuk Samsung Hospital, Sungkyunkwan University School of Medicine, Seoul, South Korea.

**Keywords:** all-cause mortality, blood pressure, cardiovascular disease mortality, hypertension, urinary albumin.

## Abstract

**BACKGROUND:**

Urinary albumin levels and hypertension (HTN) are independently associated with an increased risk of all-cause mortality. The effect of albuminuria on mortality in the absence or presence of HTN is uncertain. This study aimed to evaluate the effect of albuminuria and HTN on all-cause and cardiovascular disease (CVD) mortality.

**METHODS:**

Mortality outcomes for 32,653 Koreans enrolled in a health screening including measurements of the urinary albumin/creatinine ratio (UACR) at baseline and median follow-up of 5.13 years. Receiver operating characteristic curve analyses were performed in UACR and the cut-point was 5.42 mg/g. The participants for UACR at the cut-point of 5.42 μg/mg were categorized into UACR < 5.42 or UACR ≥ 5.42. HTN status was categorized as No HTN or HTN (defined as the absence or presence HTN).

**RESULTS:**

The median (interquartile) baseline UACRs were higher in those who died than in survivors. Subjects with a UACR ≥ 5.42 mg/g without or with HTN showed a similar increased risk for all-cause mortality and CVD mortality, even after adjusting for known CVD risk factors compared to those with no HTN/UACR < 5.42 (reference), (all-cause mortality; hazard ratio [HR] 1.48; 95% confidence interval [CI] 1.02–2.15: HR 1.47; 95% CI 0.94–2.32, respectively), (CVD mortality; HR 5.75; 95% CI 1.54–21.47: HR 5.87; 95% CI 1.36–25.29)

**CONCLUSIONS:**

The presence of urinary albumin and HTN is a significant determinant of CVD and death. Urinary albumin might be more attributable to CVD and all-cause mortality than HTN.

Hypertension (HTN) is an established risk factor of cardiovascular and all-cause mortality.^1^ However, more than half of all cardiovascular disease (CVD) occurs in individuals with mild HTN or normal blood pressure (BP, pre-HTN), despite a risk reduction with a decrease in BP levels.^2,3^ Further studies have identified other factors that may provide additional important predictive information regarding the risk of CVD.

Proteinuria and microalbuminuria are well known independent risk factors for CVD and mortality along with HTN.^4–6^ Moreover, HTN has been closely linked with a greater prevalence of proteinuria and microalbuminuria.^7^

The Prevention of Renal and Vascular End stage Disease (PREVEND) study showed that urinary albumin excretion is a predictor of all-cause mortality in the general population, and the relationship was apparent at levels of albuminuria currently considered to be normal.^8^ A recent meta-analysis documented that irrespective of the presence or absence of diabetes, CVD risk and mortality were increased with albuminuria at levels lower than that currently used to define microalbuminuria.^9^

In the Framingham Offspring Study, participants of middle-aged nondiabetic without hypertensive individuals, low levels of urinary albumin excretion well below the current microalbuminuria threshold predicted the development of CVD.^10^ Furthermore, some prospective studies indicated an association between urinary albumin excretion without threshold effects and cardiovascular mortality in the general population.^8,11^

However, it remains unclear whether urinary albumin with or without HTN predicts a risk of CVD events and death in the general population. Moreover, there are no reports that compare subjects with HTN to subjects with albuminuria below the microalbuminuria threshold level with regard to the risk of all-cause death and CVD. Recently, we reported in cohort study during a maximum of 11 years of follow-up that low grade albuminuria (below the microalbuminuria level) is an independent risk factor for incident HTN and CVD mortality.^12^

Thus, the aim of this study was to investigate the effect of HTN and urinary albumin on the risk of CVD and all-cause mortality in a large, predominantly single ethnicity, occupational Korean cohort.

In addition, the risk of death and CVD were analyzed with regard to the degree of albuminuria according to subgroups that were known to have conventional cardiovascular risk factors.

## METHODS

### Study population

The study population consisted of individuals with urinary albumin/creatinine ratio (UACR, mg/g) measurements who participated in a comprehensive health screening program at Kangbuk Samsung Hospital, Seoul, Korea, from 2002 to 2012 (*n* = 44,964). For this analysis, 12,311 subjects were excluded for one of more of the following reasons: 4,794 subjects had missing data on smoking status, alcohol consumption, and exercise; 868 subjects had a pre-existing history of malignancy; and 1 subject had an unknown vital status. Further analyses were undertaken after excluding subjects with diabetes (*n* = 3,435) and subjects with antihypertensive medication (*n* = 5,874). The total number of eligible individuals for the study was 32,653.

This study was approved by the Institutional Review Board of Kangbuk Samsung Hospital. The requirement for informed consent was waived, and deidentified information was retrieved retrospectively.

### Measurements

Data on medical history, medication use, and health-related behaviors were collected through a self administered questionnaire. Details regarding alcohol use included the frequency of intake per week. Current smokers were identified, and the weekly frequency of moderate- or vigorous-intensity physical activity was assessed. Body weight was measured with the subject in light clothing and no shoes to the nearest 0.1 kg using a digital scale. Height was measured to the nearest 0.1 cm.

Body mass index (BMI) was calculated as weight in kilograms divided by height in meters squared. Trained nurses measured sitting BP with a standard mercury sphygmomanometer.

Blood specimens were sampled from the antecubital vein after 12 hours of fasting. Serum levels of glucose, total cholesterol, triglycerides, low-density lipoprotein (LDL) cholesterol, and high density lipoprotein (HDL) cholesterol were measured using Bayer Reagent Packs (Bayer Diagnostics, Leverkusen, Germany) on an automated chemistry analyzer (Advia 1650 Autoanalyzer; Bayer Diagnostics). Insulin levels were measured with an immunoradiometric assay (Biosource, Nivelle, Belgium) with an intra- and interassay coefficient of variation of 2.1–4.5% and 4.7–12.2%, respectively. Insulin resistance was estimated using the homeostasis model assessment of insulin resistance (HOMA-IR), calculated as insulin × glucose/22.5. The serum creatinine (SCr) level was measured by means of the alkaline picrate (Jaffe) method. We used an estimation of the glomerular filtration rate (GFR) to assess the degree of kidney impairment, which was calculated using the CKD-EPI equation: eGFR = 141 × min (SCr/K, 1)a × max (SCr/K, 1)−1.209 × 0.993age × 1.018 (if female) × 1.159 (if Black), where SCr is serum creatinine, K is 0.7 for females and 0.9 for males, a is −0.329 for females and −0.411 for males, min indicates the minimum of SCr/K or 1, and max indicates the maximum of SCr/K or 1.^13^

A single morning voided urine sample was used to measure the UACR. The urinary albumin concentration was determined by immunoradiometry (Radio-immunological competition assay, Immunotech Co., Prague, Czech Republic), and the urinary creatinine concentration was measured by a modified Jaffe method. The UACR measured in a spot urine sample has been reported to be highly correlated with the 24-hour urine albumin excretion level.^14^

Abdominal ultrasonography (Logic Q700 MR; GE, Milwaukee, WI, USA) using a 3.5 MHz probe was performed in all subjects by experienced clinical radiologists, and fatty liver was diagnosed or excluded based on standard criteria, including hepatorenal echo contrast, liver brightness, and vascular blurring.^15^ HTN was defined as a systolic BP ≥ 140 mm Hg, diastolic BP ≥ 90 mm Hg, a self-reported history of HTN. Diabetes mellitus was defined as a fasting serum glucose level ≥ 126 mg/dl, a self-reported history of diabetes, or the current use of diabetic medication.^16^ Obesity was defined according to those described for Asian populations, and the BMI threshold for obesity was ≥ 25 kg/m^2^.^17^ Receiver operating characteristic curve analyses were performed to find out appropriate UACR cutoff for predicting all-cause and CVD mortality.and the cut-point was 5.42 mg/g.

Four groups were then defined as follows: (i) subjects who were below the UACR 5.42 mg/g but without HTN (no HTN/UACR < 5.42); (ii) subjects who were below 5.42 for UACR with HTN (HTN/UACR < 5.42); (iii) subjects at or above 5.42 for UACR without HTN (no HTN/UACR ≥ 5.42); and (iv) subjects at or above 5.42 for UACR with HTN (HTN/UACR ≥ 5.42)

### Ascertainment of mortality

Mortality follow-up between January 1, 2002 and December 31, 2012 was based on the nationwide death certificate data of the Korea National Statistical Office. Deaths among subjects were confirmed by matching the information to death records. Causes of death were coded centrally by trained coders using the ICD-10 classification (International Classification of Diseases, 10th revision) and ICD 00-99 codes were considered to represent cardiovascular death.

### Statistical analyses

The χ^2^-test and Student’s *t* test were used to compare the characteristics of the deceased and alive study participants at baseline. We used receiver operating characteristic curve analysis to identify the optimal UACR cutoff value for predicting all-cause and CVD mortality.

Cox proportional hazards models were used to estimate adjusted hazard ratios (HRs) and 95% confidence intervals (CIs) for all-cause and cardiovascular mortality, comparing the HTN/UACR < 5.42, no HTN/UACR ≥ 5.42, and the HTN/UACR ≥ 5.42 groups with the reference no HTN/UACR < 5.42 group. The models were initially adjusted for age, sex, treatment center, year of screening exam, smoking status, alcohol intake, regular exercise, and education level (Model 1). In Model 2: the models were further adjusted for BMI, HTN, diabetes, and history of CVD. Model 3 included adjustments for the same factors as Model 2 plus an additional adjustment for eGFR. In addition, the participants were stratified into quartiles according to UACR.

UACR quartiles were categorized as following; Q1: < 3.3 mg/g, Q2: 3.3–4.6 mg/g, Q3: 4.7–7.2 mg/g, and Q4: ≥ 7.3 mg/g. The risk of all-cause mortality and CVD mortality were analyzed with the degree of albuminuria according to sub-groups, which were based on conventional cardiovascular risk factors.

Adjustment or stratification was made for multiple confounders/effect modifiers including age, sex, BMI, eGFR, alcohol intake and exercise, educational attainment (college graduation or higher), smoking status and prior evidence of CVD.

The proportional hazards assumption was checked by examining graphs of estimated log (–log) survival. Statistical analysis was performed using Stata, version 11.2. All reported *P*-values are two tailed, and *P* < 0.05 was considered statistically significant.

## RESULTS

There were 249 deaths during the follow-up period (Table 1). Table 1 describes the baseline clinical characteristics analyzed according to the vital status at follow-up. Subjects who died were older, and a higher proportion had a history of HTN at baseline. Other conventional cardiovascular risk factors including glucose, triglycerides, and blood pressure, were also higher, and the HDL-C and proportion of those performing regular exercise was lower in subjects who died by the end of the follow-up period. At baseline, mean and median UACR were higher in the group who died during follow-up compared to survivors.

**Table 1.** Baseline characteristics of the cohort according to death at follow-up

Baseline characteristics	No death	New death	*P* value
Number	32,404	249	
Men (%)	16,701 (51.5)	178 (48.5)	
Age (years)	43.9 (11.3)	55.1 (12.4)	<0.001
BMI (kg/m^2^)	23.4 (3.1)	23.4 (3.2)	0.867
Systolic BP (mm Hg)	114.7 (14.5)	120.9 (16.4)	<0.001
Diastolic BP (mm Hg)	74.3 (9.9)	78.0 (9.8)	<0.001
Higher education (%)^a^	58.5	37.1	<0.001
Regular exercise (%)^b^	18.0	17.3	0.766
Current smoker	27.1	37.4	<0.001
Alcohol intake ≥ 20 g/day (%)	19.7	26.5	0.007
Obesity (%)	27.1	37.4	<0.001
Hypertension (%)	13.9	27.7	<0.001
History of CVD (%)	6.8	8.4	0.317
Insulin (μIU/ml)	7.97 (6.24–10.18)	7.89 (6.38–10.07)	0.272
Glucose (mg/dl)	93.1 (9.4)	94.0 (10.6)	0.102
Total cholesterol (mg/dl)	195.7 (34.9)	193.5 (35.9)	0.310
LDL-C (mg/dl)	115.4 (31.1)	110.9 (30.5)	0.023
HDL-C (mg/dl)	56.4 (13.4)	56.0 (14.3)	0.679
Triglycerides (mg/dl)	100 (70–148)	109 (81–154)	0.004
HOMA IR	1.82 (1.37–2.37)	1.85 (1.43–2.39)	0.192
Urine ACR mean (mg/g)	9.9 (35.0)	17.9 (93.6)	<0.001
Urine ACR median (mg/g)	4.65 (3.32–7.32)	5.42 (3.49–9.53)	<0.001

Data are mean (SD), median (interquartile range), or percentage. Abbreviations: ACR, albumin/creatinine ratio; BMI, body mass index; BP, blood pressure; CVD, cardiovascular disease; HDL-C, high density lipoprotein cholesterol; HOMA IR, homeostasis model assessment of insulin resistance; LDL-C, low density lipoprotein cholesterol.

^a^≥college graduate.

^b^≥ 3 time per week.

Table 2 shows the baseline characteristics across the four previously defined groups based on below or above the 75th percentile for UACR levels and the absence or presence of HTN. Differences in characteristics across the four groups were statistically significant. The median UACR levels were higher in the group that had HTN at baseline compared with the group without HTN.

**Table 2.** Baseline characteristics of study subjects by urineACR quartile

Characteristics	Overall	ACR quartiles				*P*-value
		No HTN/ACR < 75%	HTN/ACR < 75%	No HTN/ACR ≥ 75%	HTN/ACR ≥ 75%	
*N* = 32,653		17,626	1,922	10,359	2,639	
Age (years)	44.0 (11.3)	42.2 (10.6)	46.7 (10.8)	44.8 (11.9)	51.1 (11.0)	<0.001
BMI (kg/m^2^)	23.4 (3.1)	23.1 (2.9)	24.8 (3.1)	23.0 (3.3)	25.2 (3.3)	<0.001
Systolic BP (mm Hg)	114.7 (14.6)	110.6 (10.9)	133.6 (11.9)	112.1 (11.6)	139.1 (14.2)	<0.001
Diastolic BP (mm Hg)	74.3 (9.9)	71.6 (7.9)	87.8 (7.7)	72.6 (8.2)	89.4 (8.8)	<0.001
Higher education (%)^a^	58.3	64.7	54.7	53.6	40.7	<0.001
Regular exercise (%)^b^	18.0	18.1	21.2	17.0	18.5	<0.001
Current smoker(%)	27.2	31.0	35.4	20.0	23.6	<0.001
Alcohol intake ≥20 g/day (%)	19.7	20.1	33.4	14.9	26.4	<0.001
Fatty liver (%)	26.1	23.0	38.1	24.7	43.7	<0.001
Obesity (%)	28.8	25.1	45.6	26.5	50.6	<0.001
History of CVD (%)	6.85	6.34	6.45	7.38	8.49	<0.001
Insulin (μIU/ml)	2.08 (1.83–2.32)	2.05 (1.80–2.29)	2.13 (1.91–2.39)	2.08 (1.83–2.33)	2.20 (1.96–2.46)	<0.001
Glucose (mg/dl)	93.1 (3.4)	92.0 (8.8)	95.8 (9.8)	93.1 (9.5)	97.6 (10.1)	<0.001
Total-cholesterol (mg/dl)	195.7 (35.0)	192.8 (33.4)	203.2 (34.5)	195.7 (36.0)	209.5 (36.7)	<0.001
LDL-C (mg/dl)	115.3 (31.1)	114.0 (30.1)	121.6 (31.4)	114.1 (32.0)	124.6 (32.5)	<0.001
HDL-C (mg/dl)	56.4 (13.4)	56.1 (13.3)	54.0 (12.5)	57.6 (13.9)	54.9 (12.4)	<0.001
Triglycerides (mg/dl)	100 (70–148)	96 (69–140)	131 (91–183)	95 (66–143)	132 (93–193)	<0.001
HOMA IR	1.82 (1.38–2.38)	1.75 (1.32–2.27)	2.01 (1.54–2.62)	1.83 (1.37–2.40)	2.14 (1.64–2.86)	<0.001
eGFR (ml/min)	82.3 (14.3)	82.6 (13.8)	78.6 (12.1)	83.7 (15.4)	78.2 (13.5)	<0.001
Urine ACR mean (mg/g)	9.94 (35.83)	3.56 (1.00)	3.70 (0.98)	17.42 (49.60)	27.78 (73.01)	<0.001
Urine ACR median (mg/g)	4.66 (3.32–7.34)	3.54 (2.82–4.34)	3.73 (2.96–4.49)	8.18 (6.44–12.74)	10.35 (7.26–19.70)	<0.001

Data are mean (SD), median (interquartile range), or percentage. Abbreviations: ACR, albumin/creatinine ratio; BP, blood pressure; BMI, body mass index; CVD, cardiovascular disease; eGFR, estimated glomerular filtration rate; HTN, hypertension; HDL-C, high density lipoprotein cholesterol; HOMA IR, homeostasis model assessment of insulin resistance; LDL-C, low density lipoprotein cholesterol.

^a^≥college graduate.

^b^≥ 3 time per week.

Table 3 shows the age-adjusted, sex-adjusted, and fully adjusted, including eGFR, HRs for all-cause and CVD mortality according to the four baseline groups. For all-cause mortality, there was a higher HR in the no HTN/UACR ≥ 5.42 and the HTN/UACR ≥ 5.42 groups (HR 1.48, CI 1.02–2.15; HR 1.47, CI 0.94–2.32, respectively) compared with the no HTN/UACR < 5.42 group (reference group).

**Table 3.** Risk of all cause and CVD mortality according to baseline urine ACR and HTN

	Person-years	Number of events	Mortality rate (100,000 person-year)	Age-sex adjusted HR (95% CI)	Multivariate HR (95% CI)		
ACR quartiles (mg/dl)					Model 1	Model 2	Model 3
All cause mortality							
No HTN/ACR < 5.42 mg/g	85828.81	101	117.7	1.00 (reference)	1.00 (reference)	1.00 (reference)	1.00 (reference)
HTN/ACR < 5.42 mg/g	11055.4	23	208.0	1.01 (0.64–1.60)	1.24 (0.72–2.15)	1.24 (0.72–2.15)	1.22 (0.70–2.10)
No HTN/ACR ≥ 5.42 mg/g	51696.3	79	152.8	1.19 (0.88–1.61)	1.54 (1.06–2.23)	1.54 (1.06–2.23)	1.48 (1.02–2.15)
HTN/ACR ≥ 5.42 mg/g	15182.2	46	303.0	1.28 (0.89–1.84)	1.51 (0.96–2.36)	1.51 (0.97–0.37)	1.47 (0.94–2.32)
CVD mortality							
No HTN/ACR < 5.42 mg/g	85828.8	11	12.8	1.00 (reference)	1.00 (reference)	1.00 (reference)	1.00 (reference)
HTN/ACR < 5.42 mg/g	11055.4	4	36.2	1.52 (0.48–4.80)	4.06 (0.80–20.58)	3.92 (0.77–19.89)	4.13 (0.81–20.93)
No HTN/ACR ≥ 5.42 mg/g	51696.3	11	21.3	1.57 (0.67–3.70)	5.46 (1.47–20.29)	5.59 (1.50–20.85)	5.75 (1.54–21.47)
HTN/ACR ≥ 5.42 mg/g	15182.18	8	52.7	1.98 (0.77–5.09)	5.41 (1.26–23.20)	5.74 (1.33–24.72)	5.87 (1.36–25.29)

Cox proportional hazard models were used to estimate HR (hazard ratio) and 95 percentage confidence intervals (95% CIs). Model 1: adjustment for age, sex, treatment center, year of screening exam, smoking status, alcohol intake, regular exercise, education level. Model 2: Model 1 plus adjustment for body mass index, HTN, and history of CVD. Model 3: Model 2 + estimated glomerular filtration rate. Abbreviations: ACR, albumin/creatinine ratio; CVD, cardiovascular disease; HTN, hypertension.

When HRs for CVD mortality were analyzed, no HTN/ACR ≥ 5.42 and HTN/ACR ≥ 5.42 group showed a similar significantly increased fully adjusted HR compared with the reference group (CVD mortality; HR 5.75; 95% CI 1.54–21.47: HR 5.87; 95% CI 1.36–25.29). However, subjects in the HTN/UACR < 5.42 group did not show statistically significantly increased HRs compared with the reference group (HR 4.13, CI 0.81–20.93). The number of event in CVD was small.

**Figure 1.**
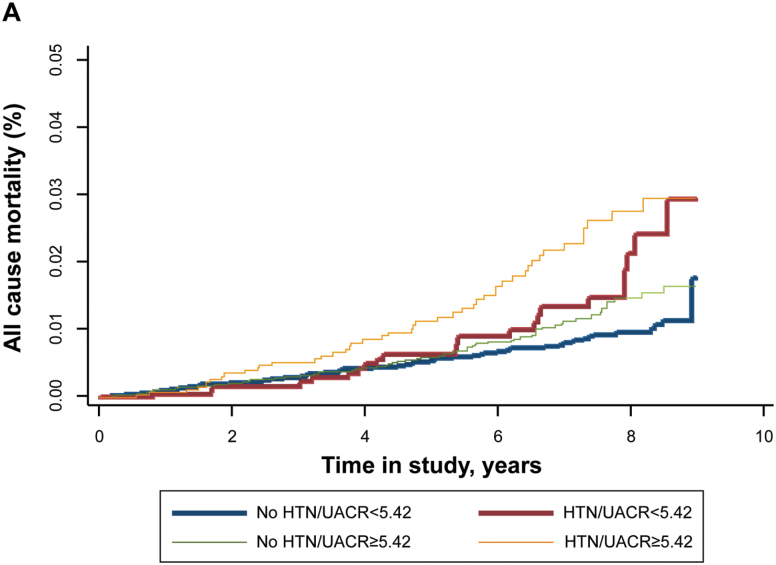

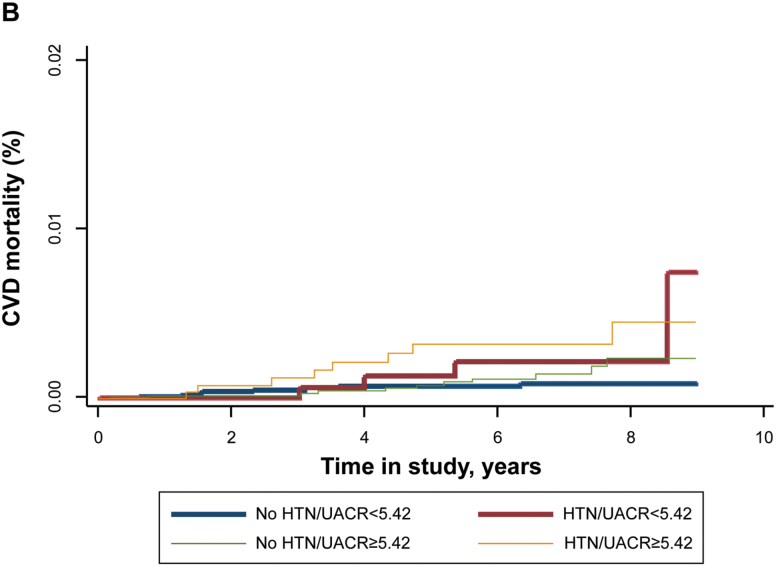
Kaplan–Meier curves for all-cause (A) all-cause death (B) CVD mortality. ACR, albumin/creatinine ratio; CVD, cardiovascular disease; HTN, hypertension. ACR < 5.42 mg/g and ≥5.42 mg/g.

Figure 1 illustrates the Kalpan–Meier curves for all-cause death(A) and CVD death(B) according to UACR < 5.42 or UACR ≥ 5.42 and HTN (hypertension) status.

Although overall mortality rates were low, subjects with a UACR ≥ 5.42 mg/g without or with HTN showed a similar increased in all-cause mortality and CVD mortality about 8 years.

The multivariate-adjusted HRs and CIs for all-cause and cardiovascular mortality according to the UACR quartiles are listed in Supplementary Tables S1 and S2.

When the HR of first quartile group for all-cause mortality was set as the reference, HRs for all-cause mortality tend to increase in proportion to the quartiles of UACR from the second to the fourth quartile group (Supplementary Table S1). However, these associations were not statistically significant.

Similar to associations with CVD mortality, the statistically significant was present in subjects with men, age ≥50 years, eGFR ≥ 90 ml/min and and group with BMI M < 25 kg/m^2^ (Supplementary Table S2).

## DISCUSSION

We describe the effects of HTN and urinary albumin levels on all-cause and cardiovascular mortality in a large number of Koreans participating in a health screening program with a median follow-up of 5.13 years.

Even small changes in the UACR (UACR level > 5.42 mg/g, below microalbuminuria level) showed an incremental association with mortality in those subjects with or without HTN. This trend was independent of eGFR levels. Importantly, for the first time, we showed that above 5.42 in UACR level in whole range of albuminuria increased risk of death and CVD to similar extent even if subject with or without HTN.

In addition, subjects with a UACR > 5.42 and HTN had a 3.27-fold increased risk of CVD morality compared to those with a UACR < 5.42 mg/g with no HTN.

In general, urinary albumin excretion is classified as: normoalbuminuria (< 30 mg per day or UACR < 30 mg/g), microalbuminuria (30–300 mg per day or UACR 30–300 mg/g, equivalent to 3.4–34 mg/mmol), and macroalbuminuria (>300 mg per day or UACR > 300 mg/g).

Furthermore, these results suggest that urinary albumin, in particular at levels below the microalbuminuria level, might be more attributable to CVD and all-cause mortality than HTN in a healthy, young, occupational cohort. Thus, this study supports the concept that any range of albuminuria as regular examination could be helpful to prevent in organ damage with hypertensive patient.

The 2013 European Society of Hypertension (ESH) and the European Society of Cardiology (ESC) hypertension guidelines recommended that all hypertensive patients have a test for microalbuminuria with a spot urine sample.^18^ In addition, the guidelines stressed that a test for microalbuminuria is a cost-effective test for organ damage in hypertensive patients.^19^ Furthermore, some prospective studies indicate that the association between urinary albumin excretion without threshold effects and cardiovascular mortality in general population^11,14^ and in non-diabetic hypertensive patients, microalbuminuria.^20^

A cohort study performed in North America, South America, and Europe that followed individuals ≥ 55 years old for a median of 4.5 years showed that any degree of albuminuria was a risk factor for the occurrence of CV events.^5^ Another multicenter cohort study involving patients with HTN and left ventricular hypertrophy also showed an association between UACR and increased cardiovascular morbidity and mortality with no UACR threshold necessary to demonstrate the increased risk.^21^ Another study suggested that urinary albumin excretion testing improves the accuracy of cardiovascular risk assessment in patients with HTN.^22^ It has also been suggested that albuminuria may be as reliable as cardiac and carotid ultrasound evaluations in predicting cardiovascular risk in hypertensive patients.^22^ Cuspidi *et al*. reported that combined ultrasound and microalbuminuria screening can improve the accuracy of target organ damage detection by 10-fold compared with routine investigations.^23^

Thus, our study suggests that a UACR > 5.42 mg/g (below the microalbuminuria level) is associated with an increased risk of death and CVD to a similar extent in those with or without HTN. This means that the UACR level, even at levels far below the microalbuminuria level, might be as useful a tool for evaluating risk stratification and target organ damage as is the presence or absence of HTN.

It is well known that overt proteinuria, macroalbuminuria, and microalbuminuria are associated with CVD mortality and other metabolic parameters.^4,11,24^ The Heart Outcomes Prevention Evaluation (HOPE) study found that there was a continuous association between albuminuria and cardiovascular events starting well below the microalbuminuria cutoff level in high risk patients with CVD.^5^ Dell’Omo *et al*. suggest the appropriateness of shifting downward the threshold level for diagnosing microalbuminuria in hypertensive patients.^25^ That study showed that high-normal albuminuria is associated with cardiovascular risk factors and predicts morbid events in hypertensive subjects. Some studies have a raised questions regarding the original definition of microalbuminuria when evaluating the risk of CVD or death.^5,13,26,27^ Thus, in recent years the focus of research has shifted towards the prognostic value of low-grade albuminuria at levels below the microalbuminuria range.^28^

The Nord-Trøndelag Health Study reported that the lowest UACR level associated with 2.2-fold increased risk for mortality was the 60th percentile (≥ 6.7 mg/g) during a 4.4-year follow-up of 2,089 subjects without diabetes and with treated HTN.^29^ In individuals with or without DM in the HOPE study, the relative risk of cardiovascular death in the fourth quartile was 1.97 (UACR > 1.62 mg/mmol) compared with the lowest quartile of the UACR.^5^ In general population with cohort study within UACR below 30mg/g, the hazard risk of HTN in fourth quartile was 1.97(UACR ≥ 7.4 mg/g) and the risk of CVD death increased 3.37-folds compared with the lowest quartile (UACR < 3.4 mg/g).^15^

Our findings provide further evidence to support the assumption that UACR, even below the microalbuminuria level (UACR cutoff > 5.42 mg/g), with or without HTN, could predict the occurrence of death as well as CVD. This result suggests that the linkage between albuminuria and mortality might not be through elevated blood pressure, which has been known to be closely associated with urinary albumin excretion in general. Thus, our study shows that the risk of death and CVD was significantly increased if the UACR was ≥ 5.42 mg/g, independent of HTN status. In addition, our study found that albuminuria is superior to HTN status in predicting all-cause and CVD mortality after adjusting for associated cardiovascular risk factors, because of the relatively short follow-up period. Although there were few cardiovascular deaths, albuminuria and HTN could have an additive effect in all-cause and CVD mortality outcome.

The pathophysiological processes that link albuminuria and CVD are unclear.

Increased albumin excretion has been related to endothelial alterations in the glomerular capillaries in patients with essential HTN.^30^ However, the cause of albuminuria in person without HTN is still uncertain. In patient without heart failure, elevated UACR predicts future hospitalization for heart failure.^31^

Increased UACR is also associated with left ventrilcular hypertrophy, a potent risk factor for progression to heart failure.^31^ Also, another researcher showed that adverse hemodynamics may play a role in albuminuria in the absence of HTN or DM.^32^

In addition, low-grade inflammation can be both a cause and consequence of endothelial dysfunction. Some previous studies have associated markers of low-grade inflammation, such as C-reactive protein, IL-6, and TNF-α, to the occurrence and progression of microalbuminuria and an increased risk for atherosclerotic disease.^33^

Alternatively, endothelial dysfunction leading stimulatneously to albuminuria and subclinical coronary artery disease (CAD) could explain the association.^34^

The multi-ethnic study of atherosclerosis including 6,774 individuals, asymptomatic individuals demonstrates an increased risk of incident coronary artery calcification (CAC) as well as greater CAC progression among those with microalbuminuria.^35^

Kramer *et al*. reported 6,814 participants without clinical CVD that mean CAC scores were higher among participants with high normal urinary albumin excretion, microalbuminuria and macroalbuminuria compared with normal urinary albumin.^34^ This study concluded that higher urinary albumin, including levels below microalbuminuria, may reflect the presence of subclinical CVD among adults without established CVD.^34^

We showed the pathologic link that increased a risk of CVD and mortality in general population may vary according to the albuminuria with the presence or absence of hypertensive status.

When interpreting our results, several limitations should be considered.

First, urinary albumin excretion was measured on a single voided urine collection, which may not have accurately reflected the true level of albuminuria. However, prior research suggested that a single-void urine UACR correlated highly with the 24-hour urinary albumin excretion with a high specificity and sensitivity; therefore, it can be used to estimate quantitative microalbuminuria.^14^

Second, we did not address the severity of HTN. Previous studies reported that BP is positively correlated with albuminuria.^12^ Thus, the level of the mean and median UACR had some differences.

Third, there is a possibility that the pharmacological therapies used by the subjects are not fully reflected in the medication history. However, prior antihypertensive medication history excluded in our study population. Thus, we excluded the influence of antihypertensive medication.

Fourth, the study was performed in a relatively homogenous population of working individuals who participated in a health screening program, and it is not fully representative of the entire Korean population. However, the number of subjects included in our study is larger than in any previous studies investigating this question.

In conclusion, this study shows that urinary albumin is an important marker for both cardiovascular and all-cause mortality in subjects with or without HTN. Subjects with a UACR ≥ 5.42 have a similar risk of death and CVD among hypertensive patients if albuminuria defined as UACR (UACR cutoff = 5.42 mg/g) is present. This increased risk is independent of age, sex, smoking status, alcohol intake, regular exercise, BMI, HTN, history of CVD, and eGFR. In the general population, the risk of death and CVD increases as the UACR increases in subjects, with or without HTN. Thus, urinary albumin is more attributable to CVD and all-cause mortality than HTN. Urinary albumin measurement might be a valuable tool in subjects with or without HTN to identify those subjects at higher risk for CVD and all-cause mortality.

## SUPPLEMENTARY MATERIAL

Supplementary data are available at *American Journal of Hypertension* online.

## DISCLOSURE

The authors declared no conflicts of interest.

## Supplementary Material

Supplementary Material
