# Supplementary material for: The Effects of Urinary Albumin and Hypertension on All-Cause and Cardiovascular Disease Mortality in Korea
Source: Am J Hypertens. 2017 May 2;30(8):799–807. doi: 10.1093/ajh/hpx051 (PMC5861583; doi:10.1093/ajh/hpx051)
Supplement: Supplementary Material [file hpx051_suppl_supplementary_material.docx]

Supplementary **1. Risk of all- cause mortality according to sub-group and quartiles of ACR concentration**

|  | **ACR quartiles(**mg/g**)** | | | | *p* for interaction |
| --- | --- | --- | --- | --- | --- |
|  | Q1(<3.3) | Q2(3.3-4.6) | Q3(4.7-7.2) | Q4(≥7.3) |  |
|  |  |  |  |  |  |
| **Men (*n*=16,879 )** |  |  |  |  |  |
| **aHR^a^ (95% CI)** | 1. (reference) | 0.87(0.52-1.47) | 0.72(0.41-1.25) | 1.25  (0.77-2.04) | 0.494 |
| **Women (*n*=15,774 )** |  |  |  |  |  |
| **aHR^a^ (95% CI)** | 1. (reference) | 0.39(0.12-1.33) | 0.69(0.24-1.97) | 0.89(0.32-2.53) |  |
| **Age <50 years (*n*=22,837)** |  |  |  |  |  |
| **aHR^a^ (95% CI)** | 1.00 (reference) | 1.74(0.79-3.84) | 1.26(0.53-3.01) | 1.50(0.64-3.48) | 0.037 |
| **Age ≥50 years (*n*=9,816)** |  |  |  |  |  |
| **aHR^a^ (95% CI)** | 1.00 (reference) | 0.49(0.26-0.94) | 0.79(0.45-1.39) | 1.40(0.85-2.31) |  |
| **Alcohol<20g/day(*n*=26,210)** |  |  |  |  |  |
| **aHR^a^ (95% CI)** | 1.00 (reference) | 0.84(0.47-1.50) | 0.82(0.46-1.45) | 1.24(0.72-2.11) | 0.962 |
| **Alcohol≥20g/day(*n*=6,443 )** |  |  |  |  |  |
| **aHR^a^ (95% CI)** | 1.00 (reference) | 0.72(0.30-1.72) | 0.71(0.29-1.71) | 0.98(0.45-2.16) |  |
| **Non orever smoker(*n*=23,791 )** |  |  |  |  |  |
| **aHR^a^ (95% CI)** | 1.00 (reference) | 0.88(0.47-1.67) | 0.72(0.38-1.38) | 1.09(0.60-1.98) | 0.389 |
| **Current smoker(*n*=8,862 )** |  |  |  |  |  |
| **aHR^a^ (95% CI)** | 1.00 (reference) | 0.59(0.28-1.26) | 0.79(0.38-1.61) | 1.20(0.63-2.30) |  |
| **Vigorous-exercise<3 time/week(*n*=26,778 )** |  |  |  |  |  |
| **aHR^a^ (95% CI)** | 1.00 (reference) | 0.78(0.46-1.33) | 0.88(0.53-1.46) | 1.17(0.72-1.89) | 0.423 |
| **Vigorous exercise≥3 time/week(*n*=5,875 )** |  |  |  |  |  |
| **aHR^a^ (95% CI)** | 1.00 (reference) | 0.83(0.26-2.67) | 0.29(0.07-1.30) | 1.18(0.37-3.74) |  |
| **<college graduate(*n*=7,286 )** |  |  |  |  |  |
| **aHR^a^ (95% CI)** | 1.00 (reference) | 0.72(0.38-1.34) | 0.72(0.39-1.33) | 1.09(0.61-1.93) | 0.859 |
| **≥college graduate(*n*=10,168 )** |  |  |  |  |  |
| **aHR^a^ (95% CI)** | 1.00 (reference) | 0.83(0.40-1.75) | 0.82(0.38-1.74) | 1.21(0.61-2.42) |  |
| **No Fatty liver (n=24,133)** |  |  |  |  |  |
| **aHR^a^ (95% CI)** | 1.00 (reference) | 0.73(0.43-1.25) | 0.72(0.42-1.24) | 1.13(0.68-1.88) | 0.912 |
| **Fatty liver (n=8,516 )** |  |  |  |  |  |
| **aHR^a^ (95% CI)** | 1.00 (reference) | 0.97(0.32-2.97) | 1.08(0.38-3.10) | 1.18(0.44-3.17) |  |
| **eGFR % <90ml/min(*n*=8,091 )** |  |  |  |  |  |
| **aHR^a^ (95% CI)** | 1.00 (reference) | 0.42(0.11-1.63) | 0.55(0.16-1.95) | 1.00(0.36-2.80) | 0.681 |
| **eGFR% ≥90ml/min (*n*=24,562 )** |  |  |  |  |  |
| **aHR^a^ (95% CI)** | 1.00 (reference | 0.88(0.52-1.48) | 0.86(0.51-1.45) | 1.18(0.72-1.94) |  |
| **No history of CVD *(n*=30,420)** |  |  |  |  |  |
| **aHR^a^ (95% CI)** | 1.00 (reference) | 0.81(0.50-1.31) | 0.79(0.49-1.28) | 1.12(0.71-1.76) | 0.873 |
| **History of CVD *(n*=2,233)** |  |  |  |  |  |
| **aHR^a^ (95% CI)** | 1.00 (reference) |  |  |  |  |
| **BMI <25 kg/m^2^ (*n*=23,241 )** |  |  |  |  |  |
| **aHR^a^ (95% CI)** | 1.00 (reference) | 0.88(0.49-1.58) | 0.81(0.45-1.48) | 1.40(0.81-2.42) | 0.795 |
| **BMI ≥25 kg/m^2^ (*n*=9,411 )** |  |  |  |  |  |
| **aHR^a^ (95% CI)** | 1.00 (reference) | 0.60(0.25-1.41) | 0.67(0.30-1.51) | 0.73(0.34-1.56) |  |

Cox proportional hazard models were used to estimate HR (hazard ratio) and 95 percent confidence intervals (95% CIs).

ACR, albumin/creatinine ratio; CVD, cardiovascular disease; HR, Hazard ratio; CI, confident interval, ; BMI, body mass index; eGFR, estimated glomerular filtration rate; Q, quartile

^a^HR, ^a^Adjustment Hazard ratio for for age, sex, center, year of screening exam, smoking status, alcohol intake, regular exercise, education level, BMI, estimated glomerular filtration rate, hypertension and history of CVD

Supplementary **2. Risk of CVD mortality according to sub-group and quartiles of ACR concentration**

|  | ACR quartiles(mg/g) | | | | *p* for interaction |
| --- | --- | --- | --- | --- | --- |
|  | Q1(<3.3) | Q2(3.3-4.6) | Q3(4.7-7.2) | Q4(≥7.3) |  |
|  |  |  |  |  |  |
| **Men (*n*=16,879 )** |  |  |  |  |  |
| **aHR^a^ (95% CI)** | 1.00 (reference) | 2.57(0.47-14.24) | 0.71(0.06-7.98) | 6.12(1.26-29.62) | <0.001 |
| **Women (*n*=15,774 )** |  |  |  |  |  |
| **aHR^a^ (95% CI)** | 1.00 (reference) |  |  |  |  |
| **Age <50 years (*n*=22,837)** |  |  |  |  |  |
| **aHR^a^ (95% CI)** | 1.00 (reference) |  |  |  | <0.001 |
| **Age ≥50 years (*n*=9,816)** |  |  |  |  |  |
| **aHR^a^ (95% CI)** | 1.00 (reference) | 0.46(0.04-5.12) | 1.27(0.20-7.94) | 3.15(0.63-15.71) |  |
| **Alcohol<20g/day(*n*=26,210 )** |  |  |  |  |  |
| **aHR^a^ (95% CI)** | 1.00 (reference) | 3.87(0.42-35.36) | 3.50(0.38-32.62) | 6.13(0.72-52.48) | 0.752 |
| **Alcohol ≥20g/day (*n*=6,443 )** |  |  |  |  |  |
| **aHR^a^ (95% CI)** | 1.00 (reference) |  | 1.59(0.09-27.52) | 2.54(0.23-28.03) |  |
| **Non or ever smoker(*n*=23,791)** |  |  |  |  |  |
| **aHR^a^ (95% CI)** | 1.00 (reference) | 1.53(0.13-17.28) | 2.62(0.28-24.71) | 3.70(0.42-32.62) | 0.886 |
| **Current smoker(*n*=8,862)** |  |  |  |  |  |
| **aHR^a^ (95% CI)** | 1.00 (reference) | 2.75(0.24-31.85) | 1.69(0.10-29.44) | 4.62(0.46-46.45) |  |
| **Vigorous exercise<3 time/week(*n*=26,778)** |  |  |  |  |  |
| **aHR^a^ (95% CI)** | 1.00 (reference) | 2.20(0.40-12.18) | 2.75(0.52-14.71) | 4.78(0.99-23.22) | 1.000 |
| **Vigorous exercise≥3 time/week(*n*=5,875)** |  |  |  |  |  |
| **aHR^a^ (95% CI)** | 1.00 (reference) |  |  |  |  |
| <college graduate**(*n*=7,286)** |  |  |  |  |  |
| **aHR^a^ (95% CI)** | 1.00 (reference) |  | 3.16(0.34-29.17) | 2.11(0.22-20.79) | <0.001 |
| **≥**college graduate**(*n*=10,168 )** |  |  |  |  |  |
| **aHR^a^ (95% CI)** | 1.00 (reference) | 6.16(0.68-56.12) |  | 10.36(1.15-93.04) |  |
| **No Fatty liver (n=24,133)** |  |  |  |  |  |
| **aHR^a^ (95% CI)** | 1.00 (reference) | 2.10(0.18-24.19) | 3.59(0.34-38.27) | 6.66(0.74-60.12) | 0.679 |
| **Fatty liver (n=8,516)** |  |  |  |  |  |
| **aHR^a^ (95% CI)** | 1.00 (reference) | 2.71(0.23-31.44) | 1.75(0.14-21.98) | 2.25(0.23-22.42) |  |
| **eGFR % <90ml/min (*n*=8,091)** |  |  |  |  |  |
| **aHR^a^ (95% CI)** | 1.00 (reference) |  |  |  |  |
| **eGFR % ≥90ml/min (*n*=24,562 )** |  |  |  |  |  |
| **aHR^a^ (95% CI)** | 1.00 (reference | 2.28(0.41-12.70) | 2.87(0.53-15.47) | 5.62(1.16-27.19) |  |
| **No history of CVD  *(n*=30,420)** |  |  |  |  |  |
| **aHR^a^ (95% CI)** | 1.00 (reference) | 2.09(0.37-11.65) | 2.43(0.45-13.18) | 3.85(0.77-19.19) | 1.000 |
| **History of CVD *(n*=2,233)** |  |  |  |  |  |
| **aHR^a^ (95% CI)** | 1.00 (reference) | 1609.74 | - | - |  |
| **BMI <25 kg/m^2^ (*n*=23,241 )** |  |  |  |  |  |
| **aHR^a^ (95% CI)** | 1.00 (reference) | 1.76(0.29-10.90) | 1.16(0.16-8.57) | 3.67(0.69-19.63) | <0.001 |
| **BMI ≥25 kg/m^2^ (*n*=9,411 )** |  |  |  |  |  |
| **aHR^a^ (95% CI)** | 1.00 (reference) |  |  |  |  |

Cox proportional hazard models were used to estimate HR (hazard ratio) and 95 percent confidence intervals (95% CIs).

ACR, albumin/creatinine ratio; CVD, cardiovascular disease; HR, Hazard ratio; CI, confident interval, ; BMI, body mass index; eGFR, estimated glomerular filtration rate; Q, quartile

^a^HR, ^a^Adjustment Hazard ratio for for age, sex, center, year of screening exam, smoking status, alcohol intake, regular exercise, education level, BMI, estimated glomerular filtration rate, hypertension and history of CVD
